# Genetic basis of anatomical asymmetry and aberrant dynamic functional networks in Alzheimer’s disease

**DOI:** 10.1093/braincomms/fcad320

**Published:** 2023-12-03

**Authors:** Nicolás Rubido, Gernot Riedel, Vesna Vuksanović

**Affiliations:** Institute of Complex Systems and Mathematical Biology, University of Aberdeen, Aberdeen AB24 3UE, UK; Institute of Medical Sciences, University of Aberdeen, Aberdeen AB25 2ZD, UK; Health Data Science, Swansea University Medical School, Swansea University, Swansea SA2 8PP, UK

**Keywords:** dynamic functional connectivity, genetic risk, cholinergic pathway, Alzheimer’s disease

## Abstract

Genetic associations with macroscopic brain networks can provide insights into healthy and aberrant cortical connectivity in disease. However, associations specific to dynamic functional connectivity in Alzheimer’s disease are still largely unexplored. Understanding the association between gene expression in the brain and functional networks may provide useful information about the molecular processes underlying variations in impaired brain function. Given the potential of dynamic functional connectivity to uncover brain states associated with Alzheimer’s disease, it is interesting to ask: How does gene expression associated with Alzheimer’s disease map onto the *dynamic* functional brain connectivity? If genetic variants associated with neurodegenerative processes involved in Alzheimer’s disease are to be correlated with brain function, it is essential to generate such a map. Here, we investigate how the relation between gene expression in the brain and dynamic functional connectivity arises from nodal interactions, quantified by their role in network centrality (i.e. the drivers of the metastability), and the principal component of genetic co-expression across the brain. Our analyses include genetic variations associated with Alzheimer’s disease and also genetic variants expressed within the cholinergic brain pathways. Our findings show that contrasts in metastability of functional networks between Alzheimer’s and healthy individuals can in part be explained by the two combinations of genetic co-variations in the brain with the confidence interval between 72% and 92%. The highly central nodes, driving the brain aberrant metastable dynamics in Alzheimer’s disease, highly correlate with the magnitude of variations from two combinations of genes expressed in the brain. These nodes include mainly the white matter, parietal and occipital brain regions, each of which (or their combinations) are involved in impaired cognitive function in Alzheimer’s disease. In addition, our results provide evidence of the role of genetic associations across brain regions in asymmetric changes in ageing. We validated our findings on the same cohort using alternative brain parcellation methods. This work demonstrates how genetic variations underpin aberrant dynamic functional connectivity in Alzheimer’s disease.

## Teaser

How do genetic variations associated with Alzheimer’s disease and the cholinergic pathway contribute to functional network involvement in the disease? In this study, Rubido *et al*., are first to report the genetic basis of anatomical asymmetry in healthy brains and its relationship with aberrant dynamic functional connectivity in Alzheimer's disease.

## Graphical Abstract

**Graphical Abstract:**
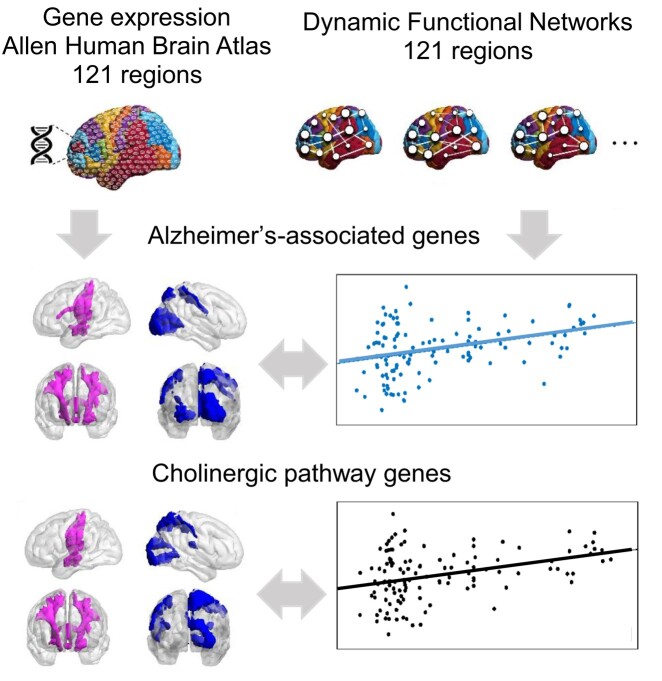

## Introduction

Alzheimer’s disease (AD) is a complex and irreversible neurodegenerative disorder, which causes cognitive impairments leading to dementia. Age is the greatest risk factor, but many other risk factors have been identified and associated with AD.^1^ For example, genome-wide association studies have identified numerous genetic variants with small cumulative effects that may explain up to 58–79% of AD inheritability and over 90% of early-onset AD.^2^ In addition, it has been suggested that the significant polygenic component of AD risk, could be ‘a valuable research tool complementing experimental designs’.^3^ Any of these genes contribute to amyloid accumulation, tau protein misfolding, the innate immune response, regulation of endocytosis, and proteasome-ubiquitin activity.^4-6^ This pathology propagates along predefined neuronal pathways.^7^ Indeed, functional brain imaging (fMRI and PET) and spatial patterns of neurodegeneration in AD mirror the anatomy of functional brain networks.^8-11^ Evidence from these studies suggests that the organization of functional networks is associated with tau protein aggregation patterns along interconnected neuronal pathways. For example, early stages of the disease present with AD-like symptoms and correlate with pathology in entorhinal and hippocampal areas, from which further cortical spread leads to increased deterioration of the patients. This selective pattern of neurodegeneration can be seen in anatomical MRI studies from AD patients,^8,12^ which have confirmed tissue loss in the entorhinal area, the hippocampus, the ventral striatum and the basal part of the forebrain in early stages of AD.^13^ The latter brain structures are well known for their high content of cholinergic neurons,^14^ with long-reaching afferents terminating in the cortical mantle and hippocampus. These projections play a critical role in learning and memory processes^15,16^ and are severely diminished in patients with AD,^17,18^ prompting the development of cholinergic treatments as a therapy. The two views are not mutually exclusive, as tau also aggregates in cholinergic cells^19^ and presumably terminals^20^ thereby compromising cholinergic tone in these target structures. Since these also comprise cortical elements, their inclusion and correlation with dynamic functional connectivity (dFC) network metrics appear warranted.

Brain network analysis from resting-state functional MRI (rs-fMRI) has advanced our understanding of the cortical organization in healthy brain.^21^ Recently, dynamic brain network analysis, which considers temporal fluctuations in the resting-state fMRI signal, has revealed patterns of activity that are usually averaged out by conventional functional network analysis. These patterns of activity, termed dynamic networks, reveal transient (metastable) dynamical states, likely involved in cognitive processing.^22^ In this context, dynamic functional networks (dFNs) analysis from fMRI data has shown a potential to unveil clinically relevant information.^21,23^ Capturing the evolving architecture of brain networks might also provide pathophysiological insights into neurological and neurodegenerative conditions, providing better diagnostic or prognostic indicators.

The non-stationary nature of the brain organization and the differences across healthy and diseased cohorts have been widely investigated by means of variations of the dwell time within different sub-network configurations.^24^ For example, a study that investigated the evolution of dFC disruptions across the AD spectrum has shown differences in patients with dementia compared to mild cognitive impairment (MCI) in terms of local dFC within the temporal, frontal-superior and default-mode sub-networks. Moreover, a decreased global metastability between functional states has also been reported.^25,26^ Consistently, studies showed a progressive loss of whole-brain metastability according to the severity of cognitive impairment along the AD continuum, reaching statistical significance only in patients with dementia when compared with healthy controls (HCs).^27,28^ These findings support the hypothesis that global patterns of brain activity in AD are progressively altered, and eventually lead to a loss of ‘dynamic complexity’ (i.e. the decrease in possible functional network’s configurations). However, neurobiological correlates of such differences have remained elusive. We therefore here explored whether functional brain networks and, in particular, their nonstationary nature are explainable by genetic association related to AD.

## Materials and methods

### Participants and cohorts

Genetic data used in this study were obtained from gene expression maps of post-mortem healthy human brains from the Allen Human Brain Atlas (AHBA) Institute.^29^ Post-mortem brains from six males and females between 18 and 68 years of age, with no known neuro-pathological history, were selected for the gene expression mapping.

Data used in the imaging part of this study were obtained from the Alzheimer's Disease Neuroimaging Initiative (ADNI) database. We downloaded demographic, clinical and MRI data from *N* = 315 participants. The main inclusion criterion was that the fMRI datasets were acquired using the same resting-state protocols (see the ‘Imaging datasets and processing’ section). The 315 participants were selected from HC, MCI and AD groups. Demographic and clinical characteristics of the participants averaged across each group are shown in Table 1.

**Table 1:** Participants’ demographic and clinical characteristics

Demographics	Healthy	MCI	AD
Number	141	128	46
Sex (F/M)	68/73	52/76	17/29
Age (SD)—years	80(6)	77(5)^a^	80(6)
Clinical scores			
MMSE	28(4)	27(3)	22(4)^a,b^

AD, Alzheimer’s disease; MCI, mild cognitive impairment; F, females; M, males; SD, standard deviation.

^a^Significant difference (*P* < 0.01) when compared with HC.

^b^Significant difference (*P* < 0.01) when compared with MCI.

### Genetic data and gene expression

Microarray gene expression data from post-mortem healthy human brains were downloaded from the AHBA.^29^ The atlas consists of 926 brain regions; each region tested using an array of 58 692 probes that correspond to 29 181 distinct genes. For the purpose of this study, the 926 AHBA regions were down-sampled to 121 regions of the Juelich Brain Atlas (JBA) (see also the ‘Construction of dynamic functional networks’ section), using the detailed anatomical labels provided by AHBA. Similarly, the 29 181 distinct genes provided by the Atlas were down-sampled to 71 genetic variants associated with Alzheimer’s disease (ADG) and 13 genetic variants associated with the cholinergic pathway (AChG). Given the involvement of the cholinergic system in the early stages of AD, we sought to address the question: How do genetic variants associated with the brain cholinergic system contribute to functional network involvement in AD?

Genes of interest were selected from The Human Protein Atlas (HPA) database.^30^ The data base generates integrated human gene-associations from curated databases and text mining. The HPA consists of 10 separate sections, each focusing on a particular aspect of the genome-wide analysis of the human proteins. We selected genes based on a query of ‘cholinergic pathway’, performed in April 2022. Using this procedure we obtained information about 16 AChG. Finally, 13 genes from the HPA database, which were also found in the AHBA gene-expression map, were used for the analysis. Similarly, 71 ADG variants were used for the part of the analysis of the ADG brain expression maps. The ADG variants were identified using 84 genes provided by Sims *et al*.^2^ and then compared with the AHBA database, resulting in down-sampled 71 variants used in the analysis. In summary, using this procedure, we obtained either 121 × 71 or 121 × 13 gene expression map for ADG and AChG variants, respectively. These maps were used for further analysis and to obtain the overall genetic map of the brain using principal component analysis (PCA).

### Statistical analysis

#### PCA of gene expression in the brain

To obtain a single vector that explains most of the variations in genetic data across brain regions, we performed a PCA on the ADG and AChG maps. We then used the first principal component for further analysis and to explore genetic associations with the functional network metrics—node strength and eigenvector centrality. We correlated the first principal component to the node strength and eigenvector centrality cortical maps. In the final step, we visualized the gene expression levels and changes in the network metrics between the groups, and we identified the regions whose gene expression level was correlated with the changes. Specifically, we visualized the relationship between gene expression in the regions and pair-wise changes in network metrics across the three groups.

### Imaging datasets and processing

We analysed rs-fMRI (or fMRI in the text) data from 315 individuals. fMRI were acquired using the ADNI-3 basic Echo Planar Imaging-blood-oxygen level-dependent (BOLD) protocol (details at ADNI imaging protocol) and in Weiner *et al.*^31^ In short, the participants had their scans taken for up to 10 min using the same two-time accelerated 3T scanners, following an even/odd interleaved axial-slicing (inferior to superior) of 3.4 mm with (3.4375 mm)^2^ pixels (FOV = 220 × 220 × 163mm; *P >> A* phase encoding; TE = 30 ms; TR = 3000 ms).

Image pre-processing, including brain extraction, registration to standard MNI space and brain tissue segmentation was carried using FMRIB’s pipeline fsl anat^32^ at its default settings. Pre-processing of fMRI was done by applying the FMRIB’s Expert Analysis Tool, FEAT, resulting in voxel-wise BOLD signals of *N*_T_ = 197 data points (197×TR acquisition times). Details on this analysis can be found in the ‘Image pre-processing details’ section of the Supplementary material.

### Construction of dynamic functional networks

Cortical regions—defining the network nodes—are based on either the Juelich (JBA)^33^ or the Harvard-Oxford Brain Atlas (H-OBA)^34^ for each participant’s fMRI. This resulted in two cortical parcellations of *N*_node_ = 121 or (48 + 21) nodes (regions/parcels), respectively. Details of the atlases given in Supplementary Table 2. JBA is a 3D atlas containing cytoarchitectonic maps of cortical areas and subcortical nuclei. The atlas is probabilistic, which enables it to account for variations between individual brains.

The signal of each node is determined by averaging fMRI BOLD signals across the regional voxels. We then defined functional links between all possible node pairs by Pearson’s correlation coefficient, *ρ*(*i, j*) (node—*i*; and node—*j*). See for example, our earlier work^22^ for details on network construction from resting-state fMRI data. We also set a 99% significance threshold to the value of each pair-wise correlation to remove spurious correlations. That is, all pairwise correlations that were not significant at *P <* 0.01 were excluded from any further analyses. This definition of functional links as a pair-wise correlation between regional BOLD time series, results in a single-subject, symmetric (undirected), weighted (with positive and negative weights), functional network that was built on statistically significant (temporal) interactions between its nodes.

In more detail, the dFN of a single participant was calculated using a sliding window approach. The exploration of window lengths (Δ*t*) on dFC networks and their characteristics, as well as choosing an optimal window-length for the analysis of fMRI data, has been described previously.^35^ Here, we used a half-overlap between consecutive sliding windows. Specifically, *t_m_* is a sliding time window defined by a multiplication factor (*m*) of the window length (Δ*t*), and the full length of the BOLD signal (*N*_T_); *t_m_* = *m*Δ*t/*2, with *m* = 0, 1*,… < N_T_/*Δ*t*, and Δ*t* = 20 data points, accounting to 1 min of scan time. As a result, *ρ*(*i, j*) changes with time, depending on the start of the sliding window. We used the significance threshold from the FC of the entire BOLD time series, given by *ρ*(*i, j*) for each participant, to threshold the dFN *ρ_t_*_Δ_(*i, j*). This corresponds to keeping sliding-window correlations that are higher than or equal to the corresponding correlations from the entire node signals (i.e. when *m* = 0 and Δ*t* = 197). Consequently, when using the HOBA and a sliding window of Δ*t* = 20 points (resulting in 18 windows over the total signal), the average per cent of links discarded was 28%, whereas when using the JBA, the average per cent of discarded links per participant was 30%.

In addition to the network analysis performed for the purpose of this study, our more detailed analysis of the dFC in the three clinical groups^36^ shows how the dFC pair-wise links evolve over the acquisition time. The analysis performed there also accounts for different network realizations, constructed on re-sampled/bootstrapped cohorts. The results show consistency across the resampled distributions. Here, we only used dFC network metrics calculated on the group’s average, to simplify the analysis and prevent any redundant results.

### Characterization of functional networks

We characterized functional connectivity in three study groups depending on the importance of the network nodes. A straightforward method of assessing the importance of a network node is to compute its centrality. The centrality of a node is a measure that quantifies how important or influential a node is within a network. Centrality can be expressed in various ways; thus, there are multiple types of centrality measures. We characterized the importance of each node in the dFNs of HC, MCI and AD individuals by calculating the node strength and eigenvector centrality. We analysed these two basic centrality network measures from the *N*_nodes_ × *N*_nodes_ correlation matrices of each participant and sliding window that is described in the section above.

#### Node strength

The simplest measure of network centrality is the so-called degree centrality, or simply node degree, which represents the number of connections a node has with other nodes in the network. In weighted networks, this becomes the node strength or weighted degree, measuring the strength of the functional connections of node *i*. For dFC networks, the node strength is calculated in each sliding window using the equation below:


(1) $$\kappa_{t\Delta }(i)=\overset{N_{\mathrm{node}}}{\underset{j=1}{\sum }}\;\rho_{t_{\Delta }}(i,j)$$

where *i* and *j* are node indices and *t*_Δ_ = *t* ∈ [*t_m_, t_m_* + Δ*t*] the sliding window. ρ_t__Δ_(*i, j*) is an element of the correlation matrix representing the strength of the pair-wise interaction between nodes *i* and *j* for the sliding window *t*_Δ_.

#### Eigenvector centrality

Eigenvector centrality is the centrality measure based on the assumption that connections to more influential nodes are more important than connections to less relevant nodes, while also taking into account the centrality of their neighbours.^37^ In simple terms, eigenvector centrality is the principal eigenvector of the network, which explains most of the variance in the data. Its principle is that links from important nodes (as measured by *κ_t_*_Δ_) are worth more than links from unimportant nodes. All nodes start off equally, but as the computation progresses, nodes with more links gain importance. Their importance also influences the nodes to which they are connected to. After recomputing many times, the values stabilize and give the final eigenvector centrality values.

Eigenvector centrality was calculated according to


(2) $$e_{t\Delta }(i)=\frac{1}{\lambda_{t\Delta }}\overset{N_{\mathrm{node}}}{\underset{j=1}{\sum }}\;\rho_{t\Delta }(i,j)e(j{)}_{t_{\Delta }}$$

where *λ_t_*_Δ_ is a constant that denotes the largest eigenvalue of *ρ_t_*_Δ_ and *e_t_*_Δ_(*i*) denotes the *i*th coordinate of the corresponding principal eigenvector; that is, a centrality score *e_t_*_Δ_(*i*) for each node *i* in an undirected network that fulfils ϱtΔe→tΔ=λtΔe→tΔ. Thus, the eigenvector centrality *e_t_*_Δ_(*i*) of a node *i* is given by the weighted sum of the values within the principal eigenvector of direct neighbours, *ρ_t_*_Δ_(*i, j*)*e_t_*_Δ_(*j*), and scaled by the proportionality factor $\lambda_{t\Delta }^{-1}$. Here, *ρ_t_*_Δ_(*i, j*) is an element of the correlation matrix representing the strength of the pair-wise interaction between nodes *i* and *j* during the sliding window *t*_Δ_ = *t* ∈ [*t_m_, t_m_* + Δ*t*], defining a time-varying vector e→tΔ  *_t_*_Δ_, whose elements are the *e_t_*_Δ_(*i*).

## Results

Our main analyses were based on two independent data sets: (i) gene expression data from six post-mortem brains and (ii) fMRI data from a sample of 315 individuals from three clinical groups: HCs, MCI and AD. dFNs were analysed across the three groups using a sliding window approach applied to the Juelich Brain Atlas regional BOLD-signal time series. The replication analysis was performed on the same subjects using another brain parcellation with a lower spatial resolution. Genetic variants associated with AD and the cholinergic brain pathway were mapped onto brain regions (of two brain atlases), and correlated with the dFC network metrics to highlight different measures of cortical organization across different modalities and scales.

### Gene expression

To test the hypothesis that the co-expression of genes of interest in the brain is associated with the differences observed in dFC between the three groups, we utilized gene expression maps from the AHBA. We used two sets of gene co-expression maps in the brain: one that consists of genes implicated with AD (71 genes) and one that consists of gene variants associated with the cholinergic brain pathways (13 genes). To reduce the dimensionality of the data we used their respective principal components, which capture the overall association patterns of the genes × brain regions maps.

**Figure 1:**
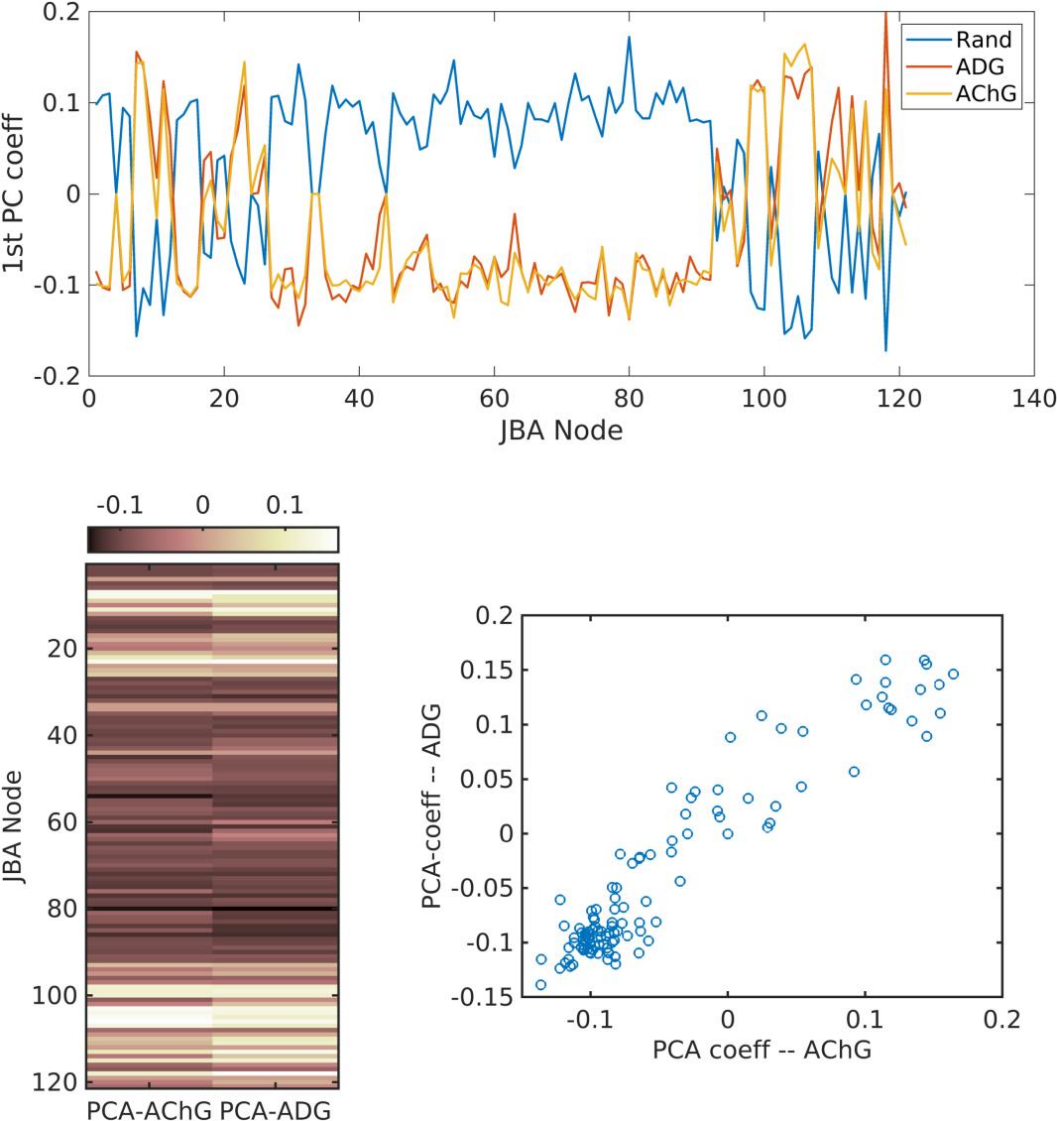
**PCA of gene co-expression across the Juelich Brain Atlas (JBA) regions.**  *Upper panel*: coefficients of the first principal component of the AChG and ADG gene covariations, and of 200 randomly selected genetic variants (Rand), within the JBA regions. *Lower panel left*: heat maps of the first principal component of the AChG and ADG gene co-variations within the JBA regions. List of regions and their ranks is given in Supplementary  Table 2. *Lower panel right*: a scatter plot of the correlation between first principal component of AChG and ADG polygenic scores for cortical associations *ρ* = 0.95 *P* << 10^–6^.

Based on the 71 genes associated with AD, we found that the first principal component of gene expression explains 51.16% of co-expression variance. Adding the additional four components explained 72.20% of total variance. Interestingly, 13 genetic variations associated with the cholinergic system in the brain explained 72.33% of co-variations across the JBA cortical parcellation (where the first five components explained 92.06% of the total variance in the data). Figure 1 depicts heat-maps of coefficients of the first principal component of AChG and ADG associations (*lower panel left*). Their correlations with the dFC measures are described in the ‘Association between functional networks and gene expression’ section.

**Figure 2:**
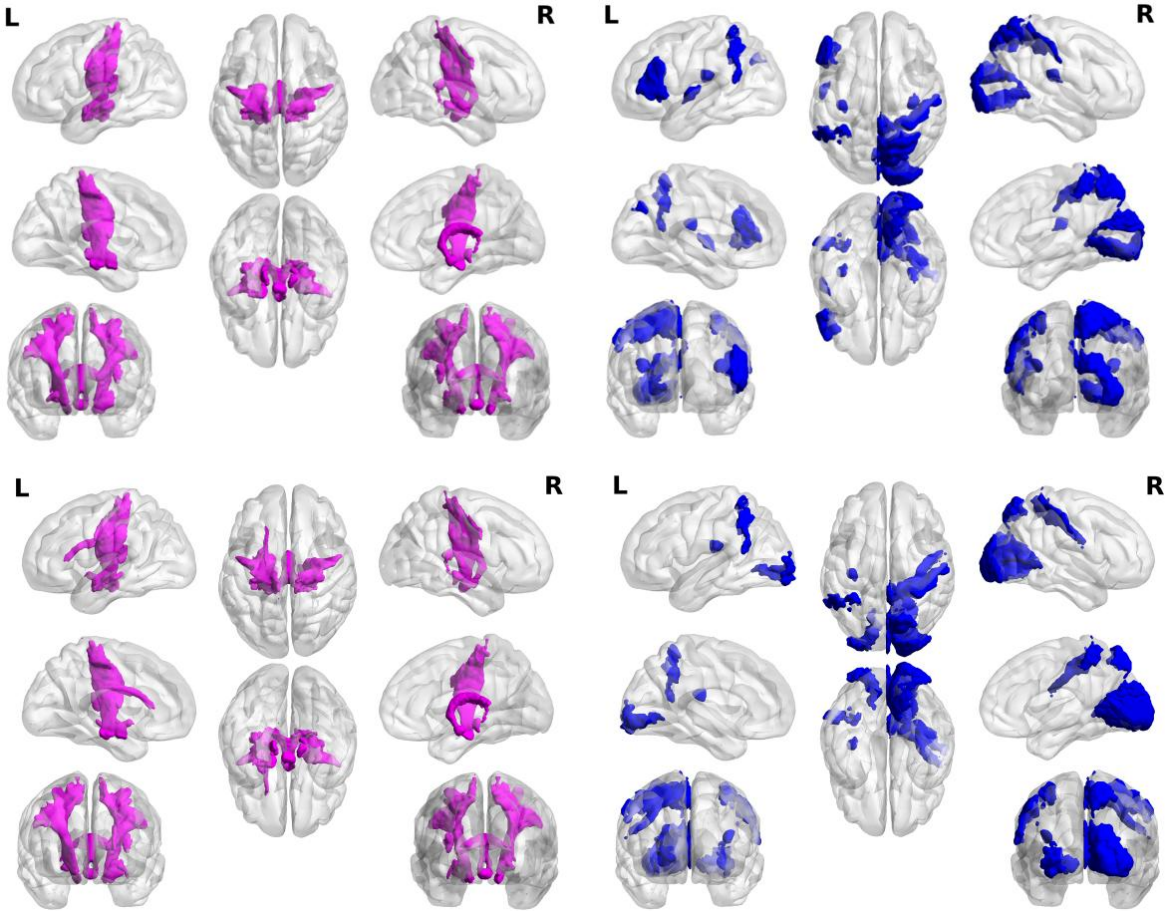
**Cortical maps of gene expression across the Juelich Atlas regions.**  *Upper panel*: the first principal component of the ACh gene co-variations with the JBA regions. *Lower panel*: the first principal component of the AD gene co-variations with the JBA regions. In both panels, cortical maps of positive coefficients of the PCA are given on the left side and negative on the right. List of regions and their ranks is summarized in Table 2. Common regions for positive coefficients: amygdala group, hippocampal-amygdaloid transition area, corticospinal tract, fornix, lateral and medial geniculate and mamillary body and insular cortex. Differential regions: uncinate fascicle and superior occipito-frontal fascicle (both found only in AD-associated-gene maps).

Figure 2 visualizes brain maps of the first principal component coefficients for ADG and AChG co-expression in JBA regions. Here, for the purpose of brevity, we show only the first 10 nodes (with the highest coefficient) for both maps. Although—visually very similar—the two maps differ in the regions mapped out (see also Table 2). Interestingly, most of the regions are spatially very close to one another. Common regions for positive coefficients: amygdala group, hippocampal-amygdaloid transition area, cortico-spinal tract, fornix, lateral and medial geniculate and mamillary body, and insular cortex. Differential regions: uncinate fascicle and superior occipito-frontal fascicle (both found only in AD-associated-gene maps).

**Table 2:** Regions of the Juelich Atlas based on positive or negative associations with gene expression of the cholinergic pathways (AChG) in the brain or Alzheimer’s disease (ADG)

AChG-positive PC coeff.	ADG-positive PC coeff.
Amygdala-centromedial group L	Amygdala-centromedial group L
Amygdala-centromedial group R	Amygdala-superficial group L
Amygdala-superficial group L	Hippocampus hippocampal-amygdaloid transition area L
Hippocampus hippocampal-amygdaloid transition area L	Corticospinal tract R
Corticospinal tract R	Corticospinal tract L
Corticospinal tract L	Fornix
Fornix	Lateral geniculate body R
Lateral geniculate body R	Lateral geniculate body L
Lateral geniculate body L	Mamillary body
Mamillary body	Medial geniculate body R
Medial geniculate body R	Superior occipito-frontal fascicle L
Medial geniculate body L	Uncinate fascicle L
Insula Ig1 L	Insula Ig1 L
**AChG-negative PC coeff.**	**ADG-negative PC coeff.**
Broca’s area BA45 L	Inferior parietal lobule PFm L
Inferior parietal lobule PFm L	Primary somatosensory cortex BA2 R
Primary auditory cortex TE1.2 L	Primary somatosensory cortex BA3a R
Primary somatosensory cortex BA2 R	Secondary somatosensory cortex/Parietal operculum OP2 L
Secondary somatosensory cortex/Parietal operculum OP2 L	Superior parietal lobule 5M R
Secondary somatosensory cortex/Parietal operculum OP2 R	Superior parietal lobule 7PC L
Superior parietal lobule 5M R	Superior parietal lobule 7P R
Superior parietal lobule 7A R	Visual cortex V1 BA17 R
Superior parietal lobule 7M L	Visual cortex V2 BA18 R
Superior parietal lobule 7PC L	Visual cortex V3V L
Superior parietal lobule 7P R	Visual cortex V3V R
Visual cortex V2 BA18 R	Visual cortex V3V R
Visual cortex V3V R	Visual cortex V3V R

First 10% ranked regions is shown.

L, left; R, right; BA, Brodmann area; PC, principal component.

The first principal component coefficients of ADG and AChG patterns of variations associated with H-OBA cortical regions is shown in Supplementary Fig. 1. Given that only sub-cortical H-OBA regions’ labelling is lateralized, separate analysis was performed on either sub-cortical or cortical regions of this atlas. The patterns of associations, in terms of their left–right asymmetry are very similar to those obtained for the JBA (see Fig. 2). It should be noted that the separate mapping of cortical and sub-cortical regions of the H-OBA showed that AChG are predominately associated with more central regions such as parahippocampus and cingulate cortices, and ADG are predominately associated with the cortical surface regions of the, e.g. occipital and temporal lobes (see Supplementary Table 1).

### Association between functional networks and gene expression

In addition to the brain-gene-expression maps, we also examined the relationship between maps and dFC metrics of interest. In particular, building on the emerging gene expression and cortical-architecture relations, we examined associations between the dFC network metrics (node strength and eigenvector centrality) and the first principal component of the ADG and AChG cortical variations. This was done by correlating the first principal components of genetic × regional co-variations with the pair-wise contrast between either the node strength or eigenvector centrality across the three groups, providing six values for each measure indicating weights of correlations.

**Figure 3:**
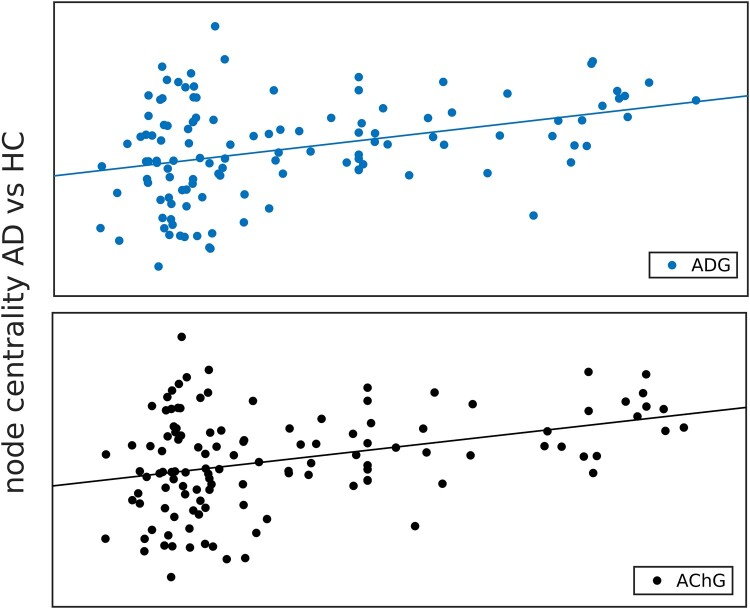
**The correlation between dFC eigenvector centrality and genetic variations.** Contrast in eigenvector centrality (z-score) between AD and HC group was correlated with the first principal component (z-score) of gene expression associated with AD (upper panel) and cholinergic system (lower panel). Correlation coefficients: rADG = 0.363 *P* < 0.0001; rAChG = 0.356; *P* < 0.0001.

Table 3 shows correlations between the first principal component and the two network measures, for the AD polygenic risk (ADG) and for the cholinergic pathway genes (AChG). As an illustration, Fig. 3 shows the correlation between AD/HC contrast for eigenvector centrality and the principal components of AChG/ADG variations. Given the high similarity in the two graphs, we also calculated the correlation between the two principal components (see Fig. 1, *lower panel left*), which reached a value of 0.95 (*P <<* 10^6^). We focused only on the first principal component, because in both cases they explain the highest portion of the variance in gene expression in the brain. We argue that the relatively high AChG co-expression in the brain (quantified by the first PC) most likely reflects a property of the JBA parcellation, which includes the superficial white matter fibres just beneath the specific cytoarchitecturally defined cortical areas. Another, equally possible, explanation is the division of the hippocampus into 10 sub-regions, where the first PC of AChG co-expression across the JBA could map cholinergic signalling in the hippocampus (see, e.g. Haam and Yakel^38^).

**Table 3:** Correlation analysis for the first principal component (*z*-score) of gene expression data and the two dFC metrics, eigenvector centrality (*z*-score) and node strength (*z*-score)

	*e*		*κ*	
	AChG	ADG	AChG	ADG
AD/MCI	0.30****	0.33****	0.27**	0.27**
AD/HC	0.36***	0.36****	0.26**	0.26**
MCI/HC	0.21*	0.16	−0.08	−0.11

*e*, eigenvector centrality; *κ*, node strength; AD, Alzheimer’s disease; MCI, mild cognitive impairment; AChG, genetic variants of the cholinergic system; ADG, genetic variants associated with AD; correlation coefficients and their respective significance values (*P*) are ****P << 1^e−5^; ***P << 1^e−4^; **P = 0.003; *P = 0.02.

### Dynamic functional networks by clinical groups

**Figure 4:**
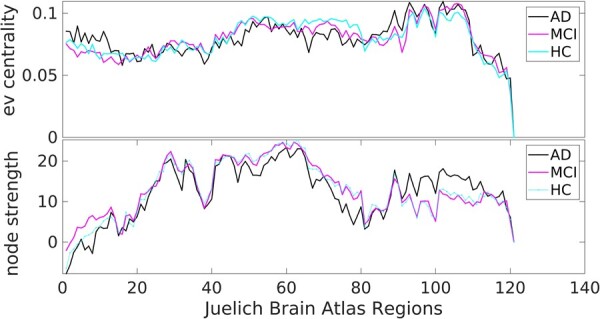
**dFC network metrics in in AD, MCI and HC groups.**  *Upper panel*: eigenvector centrality and (*lower panel*) node strength, shown when averaged over the 121 Juelich Brain Atlas regions.

Finally, we briefly describe the behaviour of the dFC networks in the three groups using the two centrality measures: the node strength and eigenvector centrality. Figure 4 shows the two network metrics across 121 regions (nodes) of the JBA, when averaged across the sliding windows. We observed statistical differences between the groups at the local (nodal) level: the dFC node strength across the white matter JBA regions differs between the AD and MCI and AD and HC groups, where the AD subjects show higher values than the other two groups. Similar statistical differences also exist for the JBA visual cortex areas, for which the AD subjects showed lower node strength than the other two groups. The eigenvector centrality revealed more ‘irregular’ differences, with the parietal and the occipital nodes’ being consistently different when comparing AD and MCI and HC subjects. A more comprehensive analysis of the dFC in the three groups^36^ showed how the fluctuations in the dFC pair-wise links evolve over the acquisition time. Here, we only show the averaged values of these measures for the purpose of visualizing their values across the JBA regions.

## Discussion

Here, we examined putative associations between genetic variations associated with AD and dFC from data of 315 individuals from three age-matched and sex-balanced clinical groups: AD, MCI and healthy seniors. Our results provide evidence of the relationship between dFC and the gene expression in the brain associated with AD, especially those co-expressed in the cholinergic pathways. We show that measures of network centrality, of any given node across the dynamic functional connections, correlate with the level of gene expression. Higher gene expression is associated with higher positive contrast in nodal strength between AD and MCI and HC, and this was most prominent in white matter regions. Lower genetic risk is associated with stronger (by absolute value) negative contrast between the AD and MCI/HC groups in the parietal and visual areas.

dFC has been identified as a better predictor of cognitive impairment in AD patients, compared to conventional static FC analysis. Numerous studies have provided compelling evidence that the classification of AD from MCI or HC subjects significantly improved when using the dFC networks as a defining feature of altered brain activity in AD.^25,39-41^ However, despite this evidence for its utility, how dFC relate to the micro-scale brain biology remains elusive. Here, we investigated dFC network metrics and genetic variations associated with AD, in an attempt to identify how large-scale network dynamics in AD relate to the microscale properties of cortical vulnerability of the disease, i.e. to variations in the regional gene expression. Our aim was to add a new genetic dimension to the differences in dFC, which has been established between AD patients and healthy subjects, yet only in terms of dynamic metastability and nodal importance in the dFC. We aimed specifically to compare, dynamic metastability and nodal strength using the parcellation of the Juelich cytoarchitectonic atlas. This is a multimodal brain atlas created to delineate white matter fibre pathways with known associated functions, as well as major white matter tracts and grey matter regions.^33^

An important question in mapping neurodegenerative processes in AD remains—how do the brain maps of gene expression relate to brain functional connectivity maps? By incorporating information about regional gene expression in the brain to annotate dFC changes in AD and MCI subjects, neural gene expression maps have been created as innovative tools explaining sources of variations in neuroimaging features across a range of neurodegenerative disorders, including Parkinsonism and dementia.^42-44^ Here, we provide the first attempt to explore this issue mapping genes and functional connectivity in the AD continuum. For this, we incorporated information about regional gene expression in the brain to annotate dFC changes and contrasted these with MCI and control subjects.

First, we produced gene expression maps onto the JBA regions, using genetic variants associated with AD (71 genes in total). Secondly, we also mapped genes associated with the cholinergic brain pathways (13 genes). This is based on the rationale that during MCI and AD there is a progressive loss of forebrain cholinergic neurons giving rise to a widespread cortical dysfunction (see Hampel *et al.*^45^ and Ferreira-Vieira *et al.*^46^ for review). Interestingly, the regions showing AD-related-polygenic associations largely overlap with those showing cholinergic pathways-related genetic associations and include: amygdala group, hippocampal-amygdaloid transition area, corticospinal tract, fornix, lateral and medial geniculate and mamillary body, and insular cortex. These maps are similar at the regional level, but show differences in the regional sub-areas and/or their inter-hemispheric homologues. While regions associated with cholinergic pathway gene co-expression are highly symmetrical and found in both hemispheres equally, regions associated with AD-related genetic variations are asymmetrically distributed across the hemispheres (see Table 2). Areas with highly emotional functions are more associated with AD genes in the left hemisphere, sensory information processing is more in the right. This is the first study of this kind to link cholinergic genetic associations with fMRI. The data therefore suggest that there is no hemisphere-selective vulnerability to cholinergic degeneration and functional loss, but rather both hemispheres are affected in the same manner in AD. Our findings are consistent with early reports on the extent of left–right symmetry in the cholinergic deficits in AD brains, which was found to be symmetrically distributed compared to more asymmetrical morphological lesions.^47,48^

Table 2 shows that it is the ADG variants which predominantly map out regions in the left hemisphere. In patients with AD, the left hemisphere is more severely affected, both structurally and metabolically.^49-51^ In addition, a trend for faster grey matter loss in the left hemisphere was also observed in age matched controls.^52^ Our results indicate that the variations in lateralization of the brain tissue loss in healthy ageing, but also in AD, may be explained by AChG and ADG variants expressed across the brain structures. As supported by early studies in AD, such loss is more symmetrical at the early stages, while becoming predominantly associated with the left hemisphere as the disease progresses.^53^ We have previously reported similar heterogeneity in brain atrophy across the cortical surface, albeit in different forms of dementia^8^ as well as in healthy ageing.^54^ This was validated across the H-OBA sub-cortical regions (see Supplementary Table 1), where 7 out of 10 regions associated with ADG variants are in the left hemisphere. However, given the differences in resolution, but also in labelling of these regions in the two atlases, our results should not be considered a 1:1 mapping at the regional level.

## Conclusion

In summary, we aimed to investigate the relationship between genetic variations and dFC in AD, MCI and healthy individuals. Linking gene expression and cortical regions provided evidence for variations in terms of hemisphere-selected vulnerability to AD. Furthermore, our findings indicated an overlap in genetic variations associated with AD and those associated with the brain cholinergic pathways; these are considered drivers of dFC aberrations in the disease. The results may provide a possible genetic substrate for changes in the metastable dynamic of cortical networks in AD, and provide evidence of genetic associations with asymmetric changes in the cortical surface in healthy ageing and AD. Our work uncovers a fundamental feature of the brain connectivity at differential scales.

## Supplementary Material

fcad320_Supplementary_Data

## Data Availability

The gene variants data used in the analyses genetic variants are available in a data repository from The Open Science Foundation (OSF). The data that support findings of this study are available from the corresponding author, upon reasonable request.
