# Supplementary material for: Genetic basis of anatomical asymmetry and aberrant dynamic functional networks in Alzheimer’s disease
Source: Brain Commun. 2023 Dec 3;6(1):fcad320. doi: 10.1093/braincomms/fcad320 (PMC10763534; doi:10.1093/braincomms/fcad320)
Supplement: fcad320_Supplementary_Data [file fcad320_supplementary_data.pdf]

Genetic basis of anatomical asymmetry and aberrant dynamic functional networks in Alzheimer's disease

Nicolás Rubido[1], Gernot Riedel[2] and Vesna Vuksanović*[3]
[1]Institute of Complex Systems and Mathematical Biology, University of Aberdeen, Aberdeen, AB24 3UE, Scotland, UK,
[2]Institute of Medical Sciences, University of Aberdeen, Aberdeen, AB25 2ZD, Scotland, UK and
[3]Swansea University Medical School, Swansea University, Data Science Building, Swansea, SA2 8PP, Wales, UK
* Corresponding author: vesna.vuksanovic@swansea.ac.uk

Supplementary Information

Image Pre-processing

fMRI pre-processing is done by FMRIB's Expert Analysis Tool (`FEAT`) with its default settings. These settings involve a high-pass filter (cut-off of at $100\,s$; $0.01\,Hz$), motion corrections (`MCFLIRT`), inter-leaved slice-timing corrections (using Fourier-space signal's phase-shifting), non-brain removal (`BET`), spatial smoothing (using a Gaussian kernel of full width-half-maximum 5 mm), and intensity normalisation. Such pre-processing is applied to all participants alike in their native space, resulting in filtered rs-fMRI with their original resolution ($3.4375 \times 3.4375 \times 3.4\,mm$) – minimising data manipulation. Quality control is carried by a visual assessment of `FEAT`'s outputs, checking that boundary-based registration (BBR) of rs-fMRI to T1w images is correctly done.

Then, a reverse normalisation of the atlas is carried. Specifically, registration of the atlas – from MNI space – to each participant's rs-fMRI native space is done by FMRIB's Linear Image Registration Tool (`FLIRT`). `FLIRT` transforms the atlas in MNI space with $2\,mm$ resolution to the native space orientation and resolution of the filtered rs-fMRI- ($3.4375 \times 3.4375 \times 3.4\,mm$). The necessary matrix transformation for this registration is obtained from `FEAT`'s rs-fMRI registration step. This step involves a BBR of the rs-fMRI to the participant's T1w high-resolution brain-extracted image, which we obtain by applying the `fsl_anat` pipeline, and a registration to the standard MNI152 brain.

Gene expression mapped onto the Harvard-Oxford Atlas regions

Validation of gene expression in the JBA regions, was performed using the Harvard-Oxford Atlas (H-OBA), one of the atlases implemented in FSL that is based on similar probability maps but with the lower resolution. The H-OBA is a probabilistic atlas covering 48 cortical and 21 subcortical structural areas, derived from structural data and segmentation, provided by the Harvard Center for Morphometric Analysis (see for example Makris *et al.,* 2006). Given that the labelling of the H-OBA, which does not differentiate left and right cortical regions, but only sub-cortical, we performed analysis on either 48 cortical or 21 sub-cortical regions separately. This was done using the same approach as described in the section Genetic Data and Gene Expression (main text).
We used two different polygenic data sets, whose variations were mapped onto the brain: one that consists of genes implicated in Alzheimer's disease (71 genes in total) and the oter one that consists of gene variants implicated in the cholinergic brain pathways (13 genes in total). To reduce the dimensionality of the genetic data, we used their respective principal components, which capture the overall association patterns genes × brain regions (for 2 brain atlases). Based on 71 genes associated with AD (or ADG in the text), we found that the first principal component explains 41.69% of co-expression variance (while the first three components have explained 66.71% variances). Similarly to the gene expression analysis for JBA regions, 13 genetic variations associated with the cholinergic system in the brain (AChG), explained 50.39% of co-variations across the JBA cortical parcellation (where the first two components explained 19.06% of the total variance in data). In the subsequent analysis of gene expression across 48 cortical and 21 subcortical regions separately, the following patterns emerged: The first principal component of AChG associations with the H-OBA regions explained 68.68% (across 48 cortical regions) and 48.89% (across 21 subcortical regions) of variance. The first principal component of ADG associations with the H-OBA regions explained 41.68% (across 48 cortical regions) and 52.22% (across 21 subcortical regions) of variance.

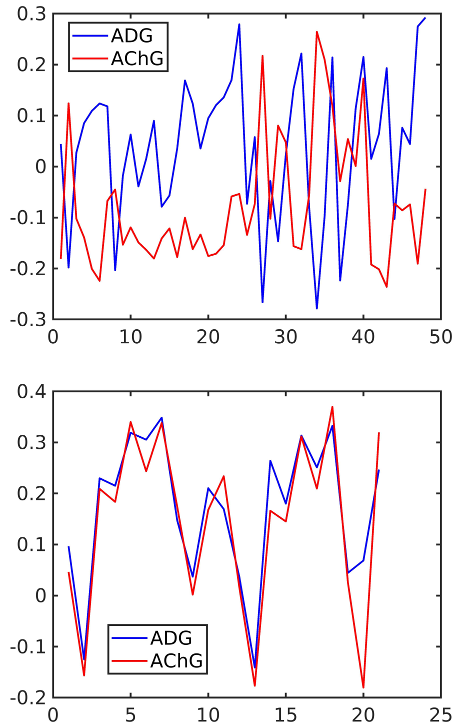

Supplementary Figure 1. First principal components of gene expression association with (upper panel) 48 cortical regions of the Harvard-Oxford Atlas and (lower panel) 21 subcortical regions of the H-O brain atlas. Abbreviations: ADG – AD-associated genes; AChG – Cholinergic-pathways genes.

Supplementary Information References

Makris, N., Goldstein, J. M., Kennedy, D., Hodge, S. M., Caviness, V. S., Faraone, S. V., ... Seidman, L. J. (2006). Decreased volume of left and total anterior insular lobule in schizophrenia. Schizophrenia research, 83(2-3), 155-171.

Supplementary Table 1. Sub-cortical and cortical regions of the Harvard-Oxford Brain Atlas which show strong positive associations with gene expression of the cholinergic pathways (AChG) in the brain or Alzheimer's disease (ADG). The first 10 regions are listed for both divisions.

ACh-positive PC coeff.	ADG-positive PC coeff.
sub-cortical hubs	
left pallidum	right pallidum
right pallidum	left caudate
left caudate	left pallidum
right caudate	right accumbens
left putamen	right caudate
right lateral ventricle	left putamen
right putamen	left accumbens
right accumbens	right putamen
left lateral ventrical	left lateral ventrical
left thalamus	left thalamus
brain-stem	left amygdala
cortical hubs	
parahippocampal gyrus, anterior	occipital pole
subcallosal	intracalcarine
parahippocampal gyrus, posterior	supracalcarine
occipital fusiform gyrus	cuneal
insular	occipital fusiform gyrus
lingual gyrus	lingual gyrus
cingulate gyrus, anterior	parietal operculum
temporal fusiform, posterior	lateral occipital, inferior
cingulate gyrus, posterior	postcentral gyrus
temporal occipital fusiform	precuneous
temporal fusiform, anterior	lateral occipital, superior

Supplementary Table 2. Juelich Brain Atlas regions. The mid lines indicate border between grey and white matter regions.

Indices	Region
1/2	Anterior intra-parietal sulcus hIP1 L/R
3/4	Anterior intra-parietal sulcus hIP2 L/R
5/6	Anterior intra-parietal sulcus hIP3 L/R
7/8	Amygdala centromedial group L/R
9/10	Amygdala laterobasal group L/R
11/12	Amygdala superficial group L/R
13/14	Broca's area BA44 L/R
15/16	Broca's area BA45 L/R
17/18	Hippocampus cornu ammonis L/R
19/20	Hippocampus entorhinal cortex L/R
21/22	Hippocampus dentate gyrus L/R
23/24	Hippocampal-amygdaloid transition area L/R
25/26	Hippocampus subiculum L/R
27/28	Inferior parietal lobule PF L/R
29/30	Inferior parietal lobule PFcm L/R
31/32	Inferior parietal lobule PFm L/R
33/34	Inferior parietal lobule PFop L/R
35/36	Inferior parietal lobule PFt L/R
37/38	Inferior parietal lobule Pga L/R
39/40	Inferior parietal lobule PGp L/R
41/42	Primary auditory cortex TE1.0 L/R
43/44	Primary auditory cortex TE1.1 L/R
45/46	Primary auditory cortex TE1.2 L/R
47/48	Primary motor cortex BA4a L/R
49/50	Primary motor cortex BA4p L/R
51/52	Primary somatosensory cortex BA1 L/R
53/54	Primary somatosensory cortex BA2 L/R
55/56	Primary somatosensory cortex BA3a L/R
57/58	Primary somatosensory cortex BA3b L/R
59/60	Sec som cortex/Parietal operculum OP1 L/R
61/63	Secondary SC/OP2 L/R
63/64	Secondary SC/OP3 L/R
65/66	Secondary SC/OP4 L/R
67/68	Superior parietal lobule 5Ci L/R
69/70	Superior parietal lobule 5L L/R
71/72	Superior parietal lobule 5M L/R
73/74	Superior parietal lobule 7A L/R
75/76	Superior parietal lobule 7M L/R
77/78	Superior parietal lobule 7PC L/R
79/80	Superior parietal lobule 7P L/R
81/82	Visual cortex V1 BA17 L/R
83/84	Visual cortex V2 BA18 L/R
85/86	Visual cortex V3V L/R
87/88	Visual cortex V4 L/R
89/90	Visual cortex V5 L/R
91/92	Premotor cortex BA6 L/R
92/93	Acoustic radiation R/L
94	Callosal body
95/96	Cingulum R/L
97/98	Corticospinal tract R/L
99	Fornix
100/101	Inferior occipito-frontal fascicle RL
102/103	Lateral geniculate body R/L
104	Mamillary body
105/106	Medial geniculate body R/L
107/108	Optic radiation R/L
109/110	Superior longitudinal fascicle R/L
111/112	Superior occipito-frontal fascicle R/L
113/114	Uncinate fascicle R/L
115/116	Insula Id1 L/R
117/118	Insula Ig1 L/R
119/120	Insula Ig2 L/R
